# Comparative Evaluation of Two Types of Immediately Loaded Implants Using Biomechanical and Histomorphometric Tests: An Animal Case Study

**DOI:** 10.5402/2012/328945

**Published:** 2012-07-18

**Authors:** Mansour Rismanchian, Bijan Movahedian, Navid Khalighinejad, Hamid Badrian, Sayed Mohammad Razavi, Afsaneh Nekouie

**Affiliations:** ^1^Dental Implant Research Center and Prosthodontics Department, Dental School, Isfahan University of Medical Sciences, Hezarjerib Street, Isfahan 8174673461, Iran; ^2^Torabinejad Dental Research Center and Oral and Maxillofacial Surgery Department, Dental School, Isfahan University of Medical Sciences, Hezarjerib Street, Isfahan 8174673461, Iran; ^3^Dental Implant Research Center, Dental School, Isfahan University of Medical Sciences, Hezarjerib Street, Isfahan 8174673461, Iran; ^4^Torabinejad Dental Research Center and Oral Pathology, Dental School, Isfahan University of Medical Sciences, Hezarjerib Street, Isfahan 8174673461, Iran; ^5^Dentistry Department, Dental School, Isfahan University of Medical Sciences, Hezarjerib Street, Isfahan 8174673461, Iran

## Abstract

*Introduction.* In order to minimize the required time to regain esthetic and function, immediately loaded implants were suggested. The aim of this study was to comparatively evaluate the Nisastan and XIve implants using biomechanical and histomorphometric tests. *Materials and Methods.* In this experimental study, 6 Nisastan one-piece immediately loaded screw type implant (OPILS) and 6 Xive implants with 3.4 mm diameter and 11 mm long were used. The implants were immediately loaded with temporary coating. After three months, the torque required to break bone-implant contact was measured and was recorded. All implants were extracted with surrounding bone and histologically were evaluated. The data were inputted into the SPSS 11.5 to run student T-test statistical analyses (*α* = 0.05). *Results.* The success rates of both types of implants was 100%, and none of them failed due to mobility or bone loss. The mean removal torque value (RTV) was 142.08 and 40 N/Cm for Xive and Nisastan implants, respectively, and their RTVs showed a significant difference between two mentioned implants (*P* = 0.004). None of the histomorphometric values showed significant differences between the two implants (*P* > 0.05). *Discussion.* both systems have the capability to induce osseointegration under immediate loads but that Xive implants showed higher capability for bone contact.

## 1. Introduction

Tooth loss especially in the esthetic zone has been a dilemma for both clinicians and patients [1]. The success rate of traditionally used implants has reached more than 95%. It has been declared that implants require from 3 to 6 months for adequate osseointegration in mandible and maxilla respectively before the second stage of the surgery [2]. Although the conventional two stage method has been successful;however, it exposes patients to increased risk of repeat surgeries and also long edentulous period. Unfortunately this long period interferes with patients' pronunciation, occlusion, and appearance with psychological consequences beyond the patients' tolerance [3].

 Due to functional and aesthetic reasons and patients' demands to overcome problems related to two stage traditional implants, technological developments have led us to meet the expectations of patients with more comfortable healing period [4, 5]. Immediate loading is defined as placing the functional occlusion on the implants not longer than 72 hours after their insertion [6–8]. It was declared that immediately loaded implants dramatically improve the psychological condition of patients compared to two-stage implants [9]. Different articles have reported promising results for immediately loaded implants as their success rate is somehow comparable with two stage conventional implants [10, 11]. In Degidi and Piattelli article [12], the success rate of immediately loaded implants was reported 93.5% after 7 years and this finding was also proved by Sennerby and Gottlow [13] as it was shown that the success rate of immediately loaded implants is comparable to conventional two-stage implants.

Immediately loaded implants' micromovements can improve osseointegration and can dramatically increase the bone density [14–17]. Also it was shown that immediate loads can increase the mineralization rate in bone-implant interface [14–17]. The rate of bone-implant contact was reported 80% with immediately loaded implants by Romanos et al. and this indicates that immediate loads improve the osseointegration [18]. Biomechanical tests are performed to assess factors like quality, strength, and stability of bone implant contact, and all of them play an important role in implants success rate [19, 20]. The most common biomechanical test to evaluate the bone-implant contact is the reverse torque test (RTT) in which a counterclockwise torque is applied to rotate the implant until the bone contact breaks [21]. *In vivo* studies have shown that reverse-torque test decrease during initial weeks, and then it increases due to bone apposition phase [22].

It should be emphasized that the type and the design of implants play an important role in the stability and the success rate of implants [23]. Xive are ADA-approved implants which are compatible with immediate loads [24–26]. Also the Iranian Nisastan implants are claimed to be similar to Xive implants structurally, and they are capable to resist occlusion loads immediately after their placement. Since there are no conclusive results regarding the capability of Nisastan implants to withstand immediate loads, this study was designed to comparatively evaluate the Nisastan and XIve implants using biomechanical and histomorphometric tests.

## 2. Methods and Materials

This was an experimental study which was held in Dentistry Faculty of Isfahan University of Medical Science aiming at comparison of bone implant contact strength between Xive (Xive, Friadent, Mannheim, Germany) and Nisastan implants (Nisastan, Isfahan, Iran) along with a histological evaluation of surrounding bone. 

## 3. Case Selection and Preparation 

Three mature healthy dogs under 2 years of age with healthy intact mandibles and teeth were selected for the experiment. In the first stage of the experiment, 10 mg/Kg Ketamine and 0.15 mg/Kg Rampone were administered intramuscularly for general anesthesia. Halothane 5% was then used to maintain the anesthesia. Using a preformed impression tray, alginate molds of the dogs' mandibular arches were prepared, master plaster impressions were cast, and temporary coatings of polymerized acrylic resin were prepared.

According to Helsinki rules, first and second premolars extraction in each quadrant was accomplished following injection of lidocaine 2% containing epinephrine 1/100,000 by infiltration in the area of premolars in order to reduce bleeding and to increase depth of anesthesia. After three months for appropriate healing of extraction site, the anesthesia procedure was performed. Dextrose saline was administered intravenously, maintaining fluids and electrolytes, and wide spectrum antibiotics including Penadore 30,000 IU/Kg and enriched sulfonamide 10 mg/Kg was administered to prevent after surgery infections. After an injection of local anesthesia, the soft tissue crestal incision was performed, and a moucoperiostal flap was elevated by a periosteal elevator, and the exact implants' sites were determined using a rotary drill. 

## 4. Implants Insertion

**Figure 1:**
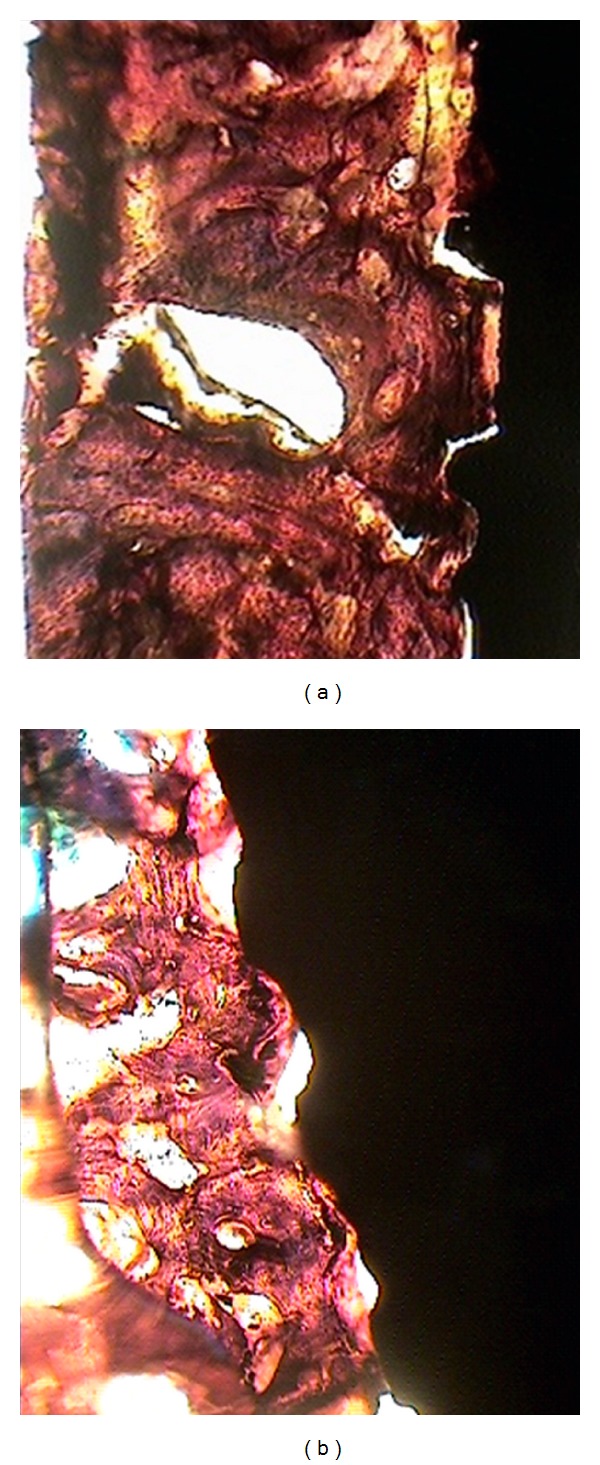
Photomicrographs illustrating used to determine bone-to-implant contact ((a) Xive implant and (b) Nisastan implant).

**Figure 2:**
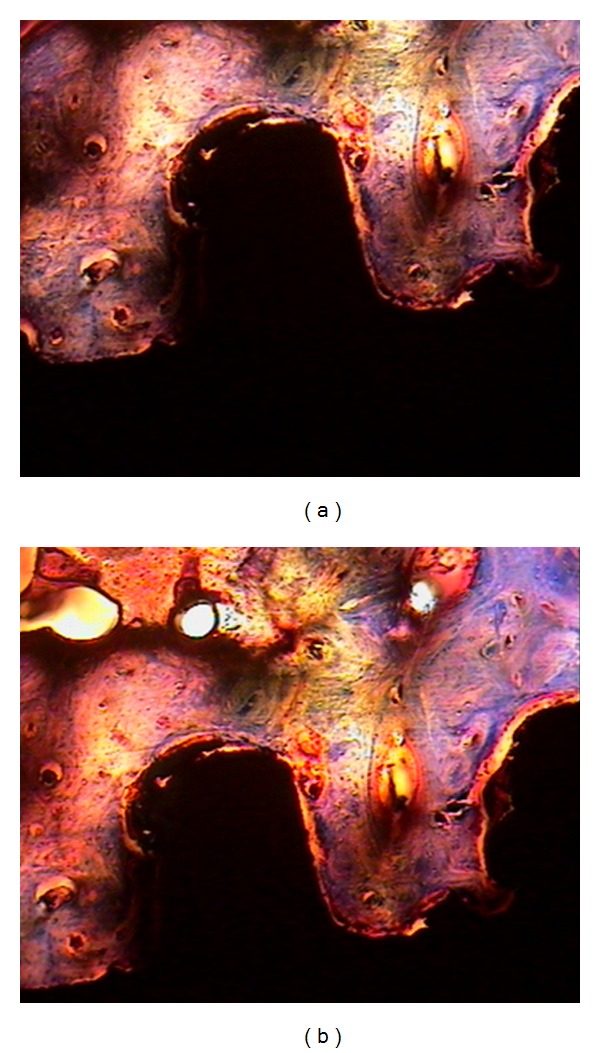
Cross-section of bone-implant surface. Bone trabeculae are in close contact with the implant surface in two groups. (a) Xive implant and (b) Nisastan implant. (Acid fuchsin and toluidine blue, optical microscope; original magnification ×100).

6 one-piece immediately loaded Nisastan screw-type implants (OPILS) and 6 Xive implants with 3.4 mm diameter and 11 mm long were used. 2 Nisastan and 2 Xive implants were inserted in the left and right side of the mandible respectively in each dog. The implants' sites in the left and right side were prepared sequential by 2, 3, and 3.4 mm diameter and 11 mm length bur (stainless steel, Institut straumann, Switzerland). All implants were screwed using Ratchet device to gain adequate primary stability according to manufacturer instruction. All implants were immediately loaded by the prepared acrylic coatings. Acrylic crowns were relined and cemented with cold cure reline acrylic resin (Meliodent; Heraeus Kulzer, Berkshire, UK) and zinc-phosphate cement (De Tray Zinc Cement, AD International limited, Weybridge, UK). The dogs were put on a soft food diet for three months while the stability of the implants and the prostheses were clinically monitored daily. At the last stage, periapical radiographs were taken to evaluate the condition of the implants and their surrounding bones. At this stage the amount of the bone loss and the stability of implants were checked. The acrylic resin coatings were then removed using crown removers. In order to perform RTT, the connecting bars were used to connect implants to fixtures and to a torque gauge (CDI Torque Products). The implants were subsequently rotated counterclockwise up to the osseointegration breakage point, and then RT values were registered. Following this, all implant samples were removed along with the surrounding bone using a 10-mm Trephine drill and immediately placed in 10% formalin solution. Once preparatory steps had been taken, the samples were carefully mounted, using a Surveyer Unit, in cold-cured acrylic resin blocks. Hard-tissue-cutting equipment (Accutom 50, Stuers, Copenhagen) was used to prepare longitudinal sections of the implants with a thickness of approximately 50 *μ*. The samples were then stained and mounted on lamella (Figure 1). Finally, the stained samples were examined under graded lenses and under a Zeiss 40x light microscope for their Bone-implant Contact (BIC), and the presence of different types of bone was recorded in percent (Figure 2). For confirmation, sample photos were reexamined using Adobe Photoshop 7.0 (San Jose, CA, USA) and values recorded in order to obtain BIC mean values from Adobe Photoshop and microscopic measurements. The data were inputted into the SPSS 11.5 to run student *t*-test statistical analyses. (*α* = 0.05).

## 5. Results

In the present prospective study, 6 immediately loaded Nisastan and 6 Xive implants were investigated. After three months the success rate of both types of implants was 100% as none of them failed due to mobility or bone loss. In the biomechanical evaluation, the minimum and maximum reverse torque values (RTVs) were 64 and 180 N/Cm in Xive and 30 and 50 N/Cm in Nisastan implants. The mean RTV was 142.08 and 40 N/Cm for Xive and Nisastan implants respectively, and the mean difference was statistically significant between two mentioned implants (*P* = 0.004).

In the histomorphometric evaluation, bone implant contact as well as extracted (either lamellar or woven) bone was measured and reported in Table 1. The point worthy of notice is that none of the above histomorphometric values showed significant differences between the two implant types (*P* > 0.05).

## 6. Discussion

The high success rate of implants changed the usual trend of prosthetic treatments. To replace a missing teeth, in order to minimize the required time to regain esthetic and function, immediately loaded implants were suggested [13, 27]. In the present study, all implants recorded 100% success rates, and this finding is in agreement with the findings of Chaushu, Degidi, Platteli, and Cooper studies [4, 28, 29].

The BIC strength was assessed using reverse torque test, and RTV revealed significant differences between two groups. Since the immediately loaded Xive implants are ADA approved, the Nisastan implants were compared with Xive to evaluate their capability. 

In the present study, the maximum and mean RTV was reported 180 and 142 N/CM, respectively, for Xive implants. In Giampiero study on 48 screw titanium implants with different machined, grit-blasted, plasma-sprayed, and acid etch surfaces, the RTV was assessed after 5 weeks. The acid-etched surface showed the higher amount of BIC strength compared to other groups but this amount was significantly lower than reported amounts for Xive implant in the present study. Xive implants are both etched and blasted surface and this can increase the surface roughness of implants and stability of implants against counter clockwise forces. Also the size of the implants used in Giampiero study was different from what we used in the present study. These could explain why the BIC strength in our study was significantly higher compared to Cordioli et al. study [30]. Also RTV in Machtei et al. study [31] on 16 pure titanium-blasted surface implants were 10 N/CM which also shows that different surface topographies can play an important role in stability and osseointegration quality of implants. Also in Newman et al. study [32], the RTV of hydroxyapatite-coated implants was higher than Xive implants in our study. It may be assumed that a molecular bond between bone and hydroxyapatite can significantly increase the RTV.

In the present study, RTV showed significant difference between Xive and Nisastan implants as the RTV was almost three times bigger in Xive implants. All implants in both groups were similar regarding their surface characteristics (acid-etched and -blasted surface). Although surface characteristics and size of the implants were similar in both groups it seems that the concentration and type of the acid and the blasting method are different in two groups, and these properties can significantly change the reaction of bone around these implants.

BIC values in both Xive and Nisastan implants under immediate loads were almost equal and around 57% (Table 1). Similar results have also been reported by Ghanavati [16], Steflik et al. [33], and Testori et al. [34]. Mechanical loading plays an important role in the preservation and maintenance of human skeleton. Wolff's Law shows the relation between mechanical factors like stress or strain and biological reactions including remodeling of bones, bone formation, or degeneration [35, 36]. It was also shown that which bones adapt themselves to loads placed on them depending on the intensity, duration, and frequency of strain as well as type and distribution forces exerted [37]. It has been reported that immediate loading of implants may increase alveolar bone density around implants [16]. Gotfredsen et al. in their study of animal implants reported higher values of BIC in implants under immediate loading than in unloaded ones [38].

The quantities of lamellar and woven bones formed within an area of 2 mm around the implant were found to be similar. For an area of 0.5 mm from implants under immediate loading, Digidi et al. [39] reported higher values of lamellar bone formation than those obtained in this study. This might be due to the longer period of loading in their study.

Although immediately loaded implants have found their niche in the field of prosthodontic treatments, their biomechanical characteristics should be assessed compared to two stage implants. So it is recommended that all ADA-approved implants for immediately loaded protocol should be investigated in a longer period of time compared to their two-stage counterparts. 

## 7. Conclusion

From the above findings, it may be concluded that the value of RTV is influenced by a variety of parameters like surface roughness, surface topography, bone site used for implant insertion, length, and diameter of implants. Furthermore, despite the fact that both Nisastan and Xive types yielded almost similar results in terms of implant stability and histological indices, bone-implant contact strength in Nisastan implants was almost one third of that in Xive implants. Given the relative similarity in the macrodesign of the two systems studied, this difference may be claimed to be due to their surface texture. Therefore, it may be concluded that both systems have the capability to induce osseointegration under immediate loads, but Xive implants represent stronger bone contact.

## Figures and Tables

**Table 1:** Bone-implant contact and composition of newly formed bone around implants.

Index	Xive		Nisastan	
	Mean	SD	Mean	SD
Degree of bone-implant contact (%)	57		57.5	
Lamellar bone formation at 2 mm (%)	60.4	3.11	60.1	2.14
Woven bone formation at 2 mm (%)	31.7	2.97	31.4	2.22

## References

[1] Aguirre-Zorzano LA, Rodríguez-Andrés C, Estefanía-Fresco R, Fernández-Jiménez A (2011). Immediate temporary restoration of single-tooth implants: prospective clinical study. *Medicina Oral, Patologia Oral y Cirugia Bucal*.

[2] Browaeys H, Defrancq J, Dierens MC A retrospective analysis of early and immediately loaded osseotite implants in cross-arch rehabilitations in edentulous maxillas and mandibles up to 7years.

[3] Brånemark PI, Hansson BO, Adell R (1977). Osseointegrated implants in the treatment of the edentulous jaw. Experience from a 10-year period. *Scandinavian Journal of Plastic and Reconstructive Surgery. Supplementum*.

[4] Chaushu G, Chaushu S, Tzohar A, Dayan D (2001). Immediate loading of single-tooth implants: immediate versus non-immediate implantation. A clinical report. *The International Journal of Oral and Maxillofacial Implants*.

[5] Cornelini R, Cangini F, Covani U, Wilson TG (2005). Immediate restoration of implants placed into fresh extraction sockets for single-tooth replacement: a prospective clinical study. *International Journal of Periodontics and Restorative Dentistry*.

[6] Anitua E, Orive G, Aguirre JJ, Andía I (2008). Clinical outcome of immediately loaded dental implants bioactivated with plasma rich in growth factors: a 5-year retrospective study. *Journal of Periodontology*.

[7] Nkenke E, Fenner M (2006). Indications for immediate loading of implants and implant success. *Clinical Oral Implants Research*.

[8] Susarla SM, Chuang SK, Dodson TB (2008). Delayed versus immediate loading of implants: survival analysis and risk factors for dental implant failure. *Journal of Oral and Maxillofacial Surgery*.

[9] Schropp L, Isidor F, Kostopoulos L, Wenzel A (2004). Patient experience of, and satisfaction with, delayed-immediate vs. delayed single-tooth implant placement. *Clinical Oral Implants Research*.

[10] Achilli A, Tura F, Euwe E (2007). Immediate/early function with tapered implants supporting maxillary and mandibular posterior fixed partial dentures: preliminary results of a prospective multicenter study. *Journal of Prosthetic Dentistry*.

[11] Degidi M, Piattelli A, Iezzi G, Carinci F (2007). Do longer implants improve clinical outcome in immediate loading?. *International Journal of Oral and Maxillofacial Surgery*.

[12] Degidi M, Piattelli A (2005). 7-year follow-up of 93 immediately loaded titanium dental implants. *The Journal of Oral Implantology*.

[13] Sennerby L, Gottlow J (2008). Clinical outcomes of immediate/early loading of dental implants. A literature review of recent controlled prospective clinical studies. *Australian Dental Journal*.

[14] Degidi M, Piattelli A (2003). Immediate functional and non-functional loading of dental implants: a 2- to 60-month follow-up study of 646 titanium implants. *Journal of Periodontology*.

[15] Calandriello R, Tomatis M, Vallone R, Rangert B, Gottlow J (2003). Immediate occlusal loading of single lower molars using Brånemark System Wide-Platform TiUnite implants: an interim report of a prospective open-ended clinical multicenter study. *Clinical Implant Dentistry and Related Research*.

[16] Ghanavati F, Shayegh SS, Rahimi H (2006). The effects of loading time on osseointegration and new bone formation around dental implants: a histologic and histomorphometric study in dogs. *Journal of Periodontology*.

[17] Romanos GE, Testori T, Degidi M, Piattelli A (2005). Histologic and histomorphometric findings from retrieved, immediately occlusally loaded implants in humans. *Journal of Periodontology*.

[18] Romanos GE, Toh CG, Siar CH, Wicht H, Yacoob H, Nentwig GH (2003). Bone-implant interface around titanium implants under different loading conditions: a histomorphometrical analysis in the Macaca fascicularis monkey. *Journal of Periodontology*.

[19] Sullivan DY, Sherwood RL, Collins TA, Krogh PH (1996). The reverse-torque test: a clinical report. *The International Journal of Oral & Maxillofacial Implants*.

[20] Meredith N (1998). Assessment of implant stability as a prognostic determinant. *The International Journal of Prosthodontics*.

[21] Albrektsson T, Sennerby L (1990). Direct bone anchorage of oral implants: clinical and experimental considerations of the concept of osseointegration. *The International Journal of Prosthodontics*.

[22] Bischof M, Nedir R, Szmukler-Moncler S, Bernard JP, Samson J (2004). Implant stability measurement of delayed and immediately loaded implants during healing. A clinical resonance-frequency analysis study with sandblasted-and-etched ITI implants. *Clinical Oral Implants Research*.

[23] Kacer CM, Dyer JD, Kraut RA (2010). Immediate loading of dental implants in the anterior and posterior mandible: a retrospective study of 120 cases. *Journal of Oral and Maxillofacial Surgery*.

[24] Rosenlicht JL (2002). SwissPlus implant system, part 1: surgical aspects and intersystem comparisons. *Implant Dentistry*.

[25] Novaes AB, de Oliveira RR, Taba M (2005). Crestal bone loss minimized when following the crestal preparation protocol: a histomorphometric study in dogs. *The Journal of Oral Implantology*.

[26] Nkenke E, Lehner B, Weinzierl K (2003). Bone contact, growth, and density around immediately loaded implants in the mandible of mini pigs. *Clinical Oral Implants Research*.

[27] Galli F, Capelli M, Zuffetti F, Testori T, Esposito M (2008). Immediate non-occlusal vs. early loading of dental implants in partially edentulous patients: a multicentre randomized clinical trial. Peri-implant bone and soft-tissue levels. *Clinical Oral Implants Research*.

[28] Degidi M, Piattelli A (2005). Comparative analysis study of 702 dental implants subjected to immediate functional loading and immediate nonfunctional loading to traditional healing periods with a follow-up of up to 24 months. *The International Journal of Oral and Maxillofacial Implants*.

[29] Cooper LF, Rahman A, Moriarty J, Chaffee N, Sacco D (2002). Immediate mandibular rehabilitation with endosseous implants: simultaneous extraction, implant placement, and loading. *The International Journal of Oral and Maxillofacial Implants*.

[30] Cordioli G, Majzoub Z, Piattelli A, Scarano A (2000). Removal torque and histomorphometric investigation of 4 different titanium surfaces: an experimental study in the rabbit tibia. *The International Journal of Oral and Maxillofacial Implants*.

[31] Machtei EE, Frankenthal S, Blumenfeld I, Gutmacher Z, Horwitz J (2007). Dental implants for immediate fixed restoration of partially edentulous patients: a 1-year prospective pilot clinical trial in periodontally susceptible patients. *Journal of Periodontology*.

[32] Newman MG, Takei HH, Carranza FA (2002). *Clinical Periodontology*.

[33] Steflik DE, Lake FT, Sisk AL (1996). A comparative investigation in dogs: 2-year morphometric results of the dental implant-bone interface. *The International Journal of Oral and Maxillofacial Implants*.

[34] Testori T, Szmukler-Moncler S, Francetti L, Del Fabbro M, Trisi P, Weinstein RL (2002). Healing of Osseotite implants under submerged and immediate loading conditions in a single patient: a case report and interface analysis after 2 months. *International Journal of Periodontics and Restorative Dentistry*.

[35] Hansson S (2003). A conical implant-abutment interface at the level of the marginal bone improves the distribution of stresses in the supporting bone: an axisymmetric finite element analysis. *Clinical Oral Implants Research*.

[36] Hansson S (1999). The implant neck: smooth or provided with retention elements—a biomechanical approach. *Clinical Oral Implants Research*.

[37] Turner CH (1998). Three rules for bone adaptation to mechanical stimuli. *Bone*.

[38] Gotfredsen K, Wennerberg A, Johansson C, Skovgaard LT, Hjørting-Hansen E (1995). Anchorage of TiO2-blasted, HA-coated, and machined implants: an experimental study with rabbits. *Journal of Biomedical Materials Research*.

[39] Degidi M, Scarano A, Piattelli M, Perrotti V, Piattelli A (2005). Bone remodeling in immediately loaded and unloaded titanium dental implants: a histologic and histomorphometric study in humans. *The Journal of Oral Implantology*.

